# Valproic acid as adjuvant treatment for convulsive status epilepticus: a randomised clinical trial

**DOI:** 10.1186/s13054-022-04292-7

**Published:** 2023-01-09

**Authors:** Tarek Sharshar, Raphaël Porcher, Pierre Asfar, Lamiae Grimaldi, Julien Jabot, Laurent Argaud, Christine Lebert, Pierre-Edouard Bollaert, Marie Line Harlay, Patrick Chillet, Eric Maury, Francois Santoli, Pascal Blanc, Romain Sonneville, Dinh Chuyen Vu, Benjamin Rohaut, Aurelien Mazeraud, Jean-Claude Alvarez, Vincent Navarro, Bernard Clair, Hervé Outin, the Valse investigators and for the Groupe d’Explorations Neurologiques en Reanimation (GENER), Laurent Argaud, Eric Azabou, François Beloncle, Omar Ben-Hadj, Pascal Blanc, Pierre-Edouard Bollaert, Francis Bolgert, Lila Bouadma, Patrick Chillet, Bernard Clair, Philippe Corne, Raphaël Clere-Jehl, Martin Cour, Arielle Crespel, Véronique Déiler, Jean Dellamonica, Sophie Demeret, Marie-Line Harley, Matthieu Henry-Lagarrigue, Julien Jabot, Nicholas Heming, Romain Hernu, Achille Kouatchet, Christine Lebert, Nicolas Lerolle, Eric Maury, Sophie Letrou, Aurélien Mazeraud, Alain Mercat, Satar Mortaza, Bruno Mourvillier, Hervé Outin, Catherine Paugham-Burtz, Marc Pierrot, Marion Provent, Benjamin Rohaut, Sylvie De La Salle, François Santoli, Maleka Schenk, Shidasp Siami, Vincent Souday, Tarek Sharshar, Romain Sonneville, Jean-François Timsit, Marie Thuong, Nicolas Weiss

**Affiliations:** 1 Neuro-Intensive Care Medicine, Anaesthesiology and ICU Department, GHU-Psychiatry and Neurosciences, Pole Neuro, Sainte-Anne Hospital, Institute of Psychiatry and Neurosciences of Paris, INSERM U1266, Université Paris Cité, Paris, France; 2 Université Paris Cité and Université Sorbonne Paris Nord, Inserm, INRAE, Center for Research in Epidemiology and StatisticS (CRESS), F-75004 Paris, France; 3 Centre d’Epidémiologie Clinique, AP-HP, Hôpital Hôtel Dieu, F-75004 Paris, France; 4 Department of Medical Intensive Care, University Hospital, Angers, France; 5 Clinical Research Unit, Assistance Publique - Hôpitaux de Paris University Paris-Saclay. Faculty of medicine, University of Versailles Saint-Quentin en Yvelines. Inserm U1018 Team Anti-infective evasion and pharmacoepidemiology, Boulogne-Billancourt, France; 6 Medical-Surgical Intensive Care Unit, CHU Felix-Guyon, Saint-Denis, La Réunion, France; 7 Service de Médecine Intensive-Réanimation, Hospices Civils de Lyon, Hôpital Edouard Herriot, Lyon, France; 8 Médecine Intensive Réanimation, Centre Hospitalier Départemental de Vendée, La Roche-sur-Yon, France; 9 CHRU-Nancy, Service de Médecine Intensive Réanimation, Université de Lorraine, 54000 Nancy, France; 10 Médecine Intensive Réanimation, Hôpital de Hautepierre, Hôpitaux Universitaires de Strasbourg, Strasbourg, France; 11 Service de Médecine Intensive - Réanimation, Centre hospitalier Léon Bourgeois, Châlons en Champagne, France; 12 Service de Médecine Intensive et Réanimation Hôpital Saint-Antoine, Paris-Sorbonne Université, Paris, France; 13 Médecine Intensive—Réanimation, Centre Hospitalier Robert Ballanger, Aulnay sous Bois, France; 14 Réanimation Médico Chirurgicale, Centre Hospitalier René Dubos, Pontoise, France; 15 Université de Paris Cité, INSERM UMR1137, Paris, France; 16 APHP Nord, Médecine Intensive – Réanimation, Hôpital Bichat—Claude Bernard, Paris, France; 17 General Intensive Care Unit, Sud-Essonne Hospital, Etampes, France; 18 Department of Neurology, Neuro-ICU & Brain institute - ICM, Pitié-Salpêtrière Hospital APHP, Sorbonne Université, Paris, France; 19 Anaesthesiology and ICU Department, GHU-Psychiatry and Neurosciences, Pole Neuro, Sainte-Anne Hospital, Perception and Memory Unit, Neurosciences Department, Institut Pasteur, Université Paris Cité, Paris, France; 20 Department of Pharmacology and Toxicology, Inserm U-1173, Raymond Poincare Hospital, AP-HP, Versailles Saint-Quentin-en-Yvelines University, Paris-Saclay University, 104 Boulevard Raymond Poincare, 92380 Garches, France; 21 AP-HP, Epilepsy Unit, Pitié-Salpêtrière Hospital, Sorbonne Université, and Paris Brain Institute, Paris, France; 22 General Intensive Care Unit, APHP, Raymond Poincaré Hospital, University of Versailles Saint-Quentin en Yvelines, Garches, France; 23 Intensive Care Unit Centre Hospitalier Intercommunal, Poissy/Saint-Germain-en-Laye, France

**Keywords:** Generalised convulsive status epilepticus, Intensive care unit, Seizure, Valproic acid

## Abstract

**Background:**

Generalised convulsive status epilepticus (GCSE) is a medical emergency. Guidelines recommend a stepwise strategy of benzodiazepines followed by a second-line anti-seizure medicine (ASM). However, GCSE is uncontrolled in 20–40% patients and is associated with protracted hospitalisation, disability, and mortality. The objective was to determine whether valproic acid (VPA) as complementary treatment to the stepwise strategy improves the outcomes of patients with de novo established GCSE.

**Methods:**

This was a multicentre, double-blind, randomised controlled trial in 244 adults admitted to intensive care units for GCSE in 16 French hospitals between 2013 and 2018. Patients received standard care of benzodiazepine and a second-line ASM (except VPA). Intervention patients received a 30 mg/kg VPA loading dose, then a 1 mg/kg/h 12 h infusion, whilst the placebo group received an identical intravenous administration of 0.9% saline as a bolus and continuous infusion. Primary outcome was proportion of patients discharged from hospital by day 15. The secondary outcomes were seizure control, adverse events, and cognition at day 90.

**Results:**

A total of 126 (52%) and 118 (48%) patients were included in the VPA and placebo groups. 224 (93%) and 227 (93%) received a first-line and a second-line ASM before VPA or placebo infusion. There was no between-group difference for patients hospital-discharged at day 15 [VPA, 77 (61%) *versus* placebo, 72 (61%), adjusted relative risk 1.04; 95% confidence interval (0.89–1.19);* p* = 0.58]. There were no between-group differences for secondary outcomes.

**Conclusions:**

VPA added to the recommended strategy for adult GCSE is well tolerated but did not increase the proportion of patients hospital-discharged by day 15.

*Trial registration* No. NCT01791868 (ClinicalTrials.gov registry), registered: 15 February 2012.

**Supplementary Information:**

The online version contains supplementary material available at 10.1186/s13054-022-04292-7.

## Introduction

Generalised convulsive status epilepticus (GCSE) is a diagnostic and therapeutic emergency and is defined as a convulsive seizure lasting more than 5 min, or as consecutive seizures without recovery of consciousness between seizures [1]. At the time of the design of the current trial, stepwise anti-epileptic therapy was recommended, consisting of a benzodiazepine (i.e. lorazepam, clonazepam, diazepam, or midazolam) and, if GCSE was not controlled, a second-line anti-seizure medicine (ASM), such as intravenous phenytoin/fosphenytoin, valproic acid (VPA), phenobarbital, or levetiracetam [2]. Despite this strategy, established GCSE progressed towards refractory GCSE in 20–43% of cases [3–6]. Refractory GCSE was then reported to be associated with increased in-hospital mortality (which could be high as 40% [3, 4]), increased length of hospital stay [3], only a 20% rate of return to basal clinical condition, and a 50% rate of functional disability at 90 days. In addition, a super-refractory status could develop in about 10% of cases [7]. Overall, 30–60% of patients with GCSE had then to be referred to an intensive care unit (ICU) for prolonged hospitalisation [8, 9], which was itself associated with higher mortality, long-term disability, and cognitive impairment [5, 6]. From a European registry conducted between 2011 and 2015, it was later reported that a second-line ASM was successful in only 46% of GCSE patients [10].

Experts therefore considered that interventions should be proposed as treatment adjuncts to the recommended first- and second-line ASMs, for better controlling the epileptic process (namely, anti-epileptic activity) and to improve recovery (i.e. neuroprotective activity) in GCSE patients admitted to the ICU, as these represent GCSE cases at high risk of poor outcomes [2]. One randomised clinical trial was conducted on therapeutic hypothermia in ICU-admitted GCSE patients, which found no improvement in neurological outcome and was associated with serious adverse events [6]. Based on similar considerations, we thought that addition of VPA to the second-line ASMs could be a relevant option because of its anti-epileptic, neuroprotective properties and relatively good tolerability, acknowledging that it can be associated with hyperammonemia-related encephalopathy. The neuroprotective effect of VPA involves epigenetic mechanisms [11] but also anti-inflammatory [12] and anti-NMDAR effects[13]**.** It has been evidenced or hypothesised in neurodegenerative disease [14], stroke [15], brain tumour [16, 17], epilepsy [11], and spinal cord injury [18]. Moreover, French guidelines do not recommend VPA as a second-line ASM [2], which was prescribed in GCSE patients by only 16% of physicians [19] in less than 10% of GCSE cases [10], thus allowing its administration as an adjunctive ASM.

We conducted a multicentre, double-blind, randomised controlled, and pragmatic trial to assess whether the addition of intravenous VPA to the recommended stepwise anti-epileptic strategy in patients admitted to the ICU for GCSE, would increase the number of living patients discharged from hospital by day 15 after GCSE onset [20].

## Methods

### Study design

VALSE (VALproic Acid in Status Epilepticus) is a multicentre, in parallel, randomised double-blind, controlled trial conducted in 16 French ICUs. It compared the addition of intravenous VPA with placebo in patients admitted to the ICU for GCSE, as well as to first- and second-line ASMs and standard ICU care. The overall study duration for each participant was 3 months. The trial protocol has been published previously and is available with the full text of this article. [20].

Sixteen centres, including 8 general hospitals and 8 university hospitals, participated in this study. Training on study procedures was provided to all participating staff members.

### Eligibility criteria

Adult patients were eligible if admitted to the ICU for GCSE, defined by 5 min or more of continuous or recurrent generalised convulsive seizure without recovery of consciousness between seizures [1, 2]. The clinical seizures could have ceased or not and consciousness could be impaired or not at time of inclusion. In all cases, however, the anti-epileptic treatment (i.e. first- and/or second-line ASM) should have been initiated within the 6 h prior to inclusion (Additional file 1: Appendix).

Main exclusion criteria were non-convulsive status epilepticus clinically characterised by altered mental status but with no motor symptoms at any time during the course of status epilepticus [1], post-anoxic status epilepticus, previous treatment by VPA prior to randomisation, hospitalisation for a disease associated with an expected length of stay > 15 days, expected ICU length of stay < 12 h, life expectancy < 3 months, women of childbearing age (> 17 and < 50 years), VPA contraindications (specifically, acute and chronic hepatitis, Child B or C cirrhosis), previous enrolment in an interventional trial including the VALSE trial, absence of health insurance coverage, and under guardianship. Thus, cases of GCSE that progressed to non-convulsive status epilepticus (also called *subtle status epilepticus*) were not excluded (Additional file 1: Appendix).

All patients admitted for GCSE in one of the participating ICUs were screened for eligibility by the ICU physicians and reasons for non-randomisation were collected.

### Randomisation and interventions

Eligible patients were randomly assigned in a 1:1 ratio to receive either VPA or placebo. Randomisation, with stratification according to site, age, and presence of acute brain injury, was performed with the use of a central concealed, Web-based, automated randomisation system. VPA treatment consisted of intravenous administration of a loading dose of 30 mg/kg over 15 min followed by a continuous intravenous dose of 1 mg/kg/h over the next 12 h [21]. Placebo comprised an identical intravenous administration of 0.9% saline as a bolus and continuous infusion.

In both groups, the anti-epileptic treatment was standardised. If not initiated before ICU admission, the patients had to receive the first- and second-line ASMs before the administration of VPA. More specifically, in patients who had not received a second-line ASM before their admission to the ICU, the second-line ASM was to be administered when the GCSE evolving over 5 to 30 min had required several intravenous boluses of benzodiazepines, or when the seizures were controlled by a single administration of benzodiazepines but beyond 30 min after their onset, as recommended by the 2009 French Expert Guidelines [2]. This clause allowed for the inclusion primarily of de novo established GCSE (Additional file 1: Appendix). According to national guidelines [2], the first-line ASM included clonazepam or diazepam; second-line ASMs included phenobarbital, fosphenytoin or levetiracetam. Recommended ASM for refractory and super-refractory GCSE included infusion of sedating agents (i.e. propofol or midazolam) and thiopental, respectively [2]. Maintenance ASM was decided by the local physician, independently of the trial protocol. In both groups, patients received standardised care, including control of secondary brain injuries (temperature, mean blood pressure, blood glucose, sodium levels, PaO_2_ and PaCO_2_ regulation), aetiological investigations, and neurological monitoring [22]**.**

### Outcomes and assessment

The primary outcome was the proportion of living patients discharged from hospital to their home or to a long-term care facility on day 15. The primary endpoint (i.e. hospital status at day 15) was collected by a local investigator, blinded to group assignment (Additional file 1: Appendix).

Secondary outcomes were recurrence of seizure during ICU stay, occurrence of refractory and super-refractory GCSE during ICU stay, occurrence of adverse events whilst in hospital, and cognitive status at day 90 assessed with the Mini-Mental State Examination (MMSE), Frontal Assessment Battery (FAB), and Glasgow Outcome Scale-Extended (GOSE). Follow-up visits occurred at day 15, at ICU and hospital discharge, and at day 90.

Patients were assessed neurologically every 4 h whilst in the ICU, notably for awakening, focal neurological signs, and abnormal movements. In both groups, serum samples were obtained prior to, and 15 min and 12 h after, administration of the study drug load in order to measure serum VPA concentrations. Samples were stored at − 20 °C in the participating centres before being sent to the Department of Pharmacology and Toxicology of the Raymond Poincaré Teaching Hospital (Garches, France) for centralised VPA measurements. In every patient, administration of a standard EEG was recommended within 24 h of ICU admission and again between day 2 and day 7. EEGs were interpreted by the referring neurophysiological team of the participating centre, which was blinded to study drug groups.

Up to hospital discharge, we collected the time to awakening, length of ICU and hospital stays, in-ICU and in-hospital mortality, and changes in the maintenance ASMs. At day 90, the referring neurologist or intensivist assessed MMSE and FAB through a medical examination and the GOSE by phone.

### Statistical analysis

The study was powered to detect an absolute increase of 20% in the rate of patients discharged alive at day 15 with a power of 90% and a two-sided 0.05 significance level, assuming a 50% rate in the control group. Accordingly, the sample size was 124 patients per group. To account for potential errors in the administration of the allocated treatment, this number was increased to 150 per group.

The analysis followed the intention to treat (ITT) principle, and all randomised participants were analysed in the group allocated by randomisation, regardless of the actual treatment received or other protocol deviations. Only participants withdrawing their consent during the study and opposing analysis of their data were excluded from the analysis. The Statistical Analysis Plan was developed blinded to treatment assignment (i.e. without knowledge of group allocation), except for the last amendment concerning fallback solutions when some models did not converge. When drafting the first version of the Statistical Analysis Plan, some changes were made compared to the original protocol: (1) the last observation carried forward approach to handle supplemented by multiple imputation for missing data, given that the primary outcome was missing for two participants only; (2) the analytic model for binary outcomes changed from logistic regression with random centre effects to a log-binomial model with robust standard errors in order to directly estimate relative risks (RRs) instead of odds ratios and limit issues with convergence of binomial generalised linear mixed models [23, 24]. Data analysis was masked to the actual treatment.

Missing primary outcome data were treated as failures, and sensitivity analyses with the worst-case scenario imputation (imputing a failure in the experimental group but a success in the control group) or available data analysis were carried out. No imputation was performed for secondary efficacy and safety outcomes.

Binary outcomes were analysed with a log-binomial model adjusted for the randomisation strata, with robust standard errors to account for centre [25]. Adjusted risk differences were derived using regression standardisation [26]. Time-to-event data were analysed using Cox or Fine-Gray regression models, the latter when death was a competing risk [27], all models being adjusted for the randomisation stratification variable and with random centre effects.

No correction for multiplicity and no hierarchical testing procedures were used in analysing secondary outcomes, which should be regarded as exploratory. Analyses were performed using R 4.0.5 software (The R Foundation for Statistical Computing, Vienna, Austria).

## Results

**Fig. 1:**
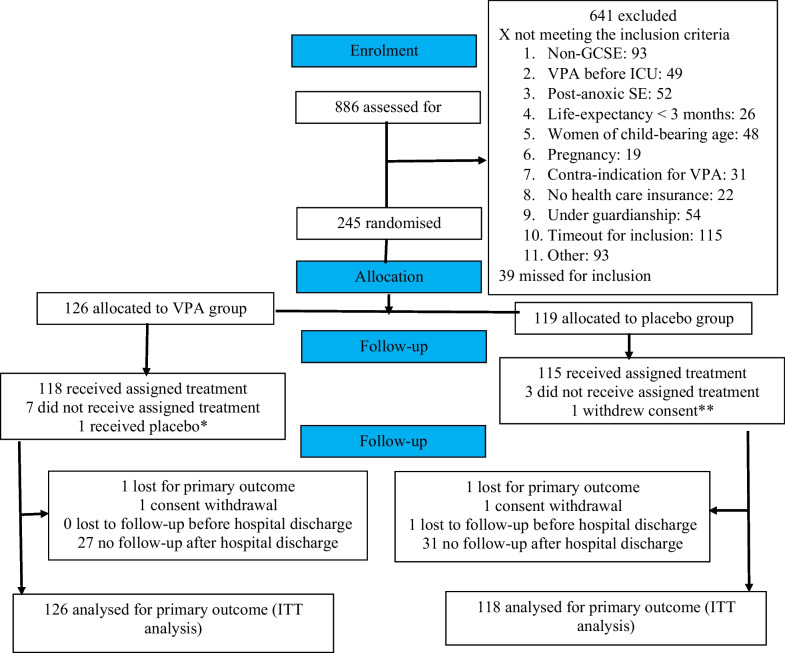
Study Profile. * Error in distributing the allocated blinded treatment. ** Per participant request, all data concerning this individual have been erased from the trial database. *GCSE* Generalised convulsive status epilepticus; *ICU* Intensive care unit; *ITT* Intention to treat; *VPA* Valproic acid

Participants were enrolled between 18 February 2013 and 5 July 2018 when, because of difficulties in recruitment, the sponsor made the decision to discontinue the study. A total of 245 patients were enrolled, one of whom withdrew consent. 244 patients were therefore included in the main analysis, 126 (52%) in the VPA (i.e. intervention) and 118 (48%) in the placebo (i.e. control) groups (Fig. 1). One patient assigned to the VPA group received placebo. Three patients in the placebo group and 7 in the VPA group did not receive the assigned treatment.

The baseline characteristics of the patients were similar between the two groups (Table 1 and Additional file 1: Table S1). The time from seizure onset to ICU admission was a median of 2.7 h (interquartile range (IQR): [2.0–3.8]). At ICU admission, 224 (93%) patients received benzodiazepines as first-line ASM and 141 (58%) as second-line ASM. All patients, who were in refractory GCSE at time of their admission in ICU, had then received a sedating ASM. A total of 165 (68%) patients received invasive mechanical ventilation at time of admission and 41 (17%) additional patients were intubated within the first 24 h. Status epilepticus was refractory in 22 (9%) cases at time of ICU admission. 168/238 (71%) participants received a 30-min EEG within 24 h after ICU admission and 83/201 (41%) between 2 and 7 days, evidencing persisting seizures in 17/168 (10%) and 6/83 (7%) patients, respectively.

**Table 1:** Characteristics of the patients at randomisation

Characteristics	*n*	Placebo (*n* = 118)	*n*	Valproic acid (*n* = 126)
Age, median (IQR) year	118	56.2 (44.7–68.1)	126	58.5 (44.8–68.6)
> 65 year—no. (%)	118	41 (35)	126	44 (35)
Male sex—no. (%)	118	76 (64)	126	84 (67)
*Medical history*				
History of epilepsy—no. (%)	117	57 (49)	123	60 (49)
Pre-existing anti-epileptic treatment—no. (%)	117	50 (43)	123	59 (48)
Other pre-existing neurological disease—no. (%)	117	64 (55)	123	71 (58)
*Characteristics of the status epilepticus at randomisation*				
Acute brain injury—no. (%)	118	17 (14)	126	28 (22)
Seizure type—no. (%)	115		123	
Primary generalised status epilepticus		97 (84)		89 (72)
Secondary generalised status epilepticus		18 (16)		34 (28)
Focal neurological signs—no. (%)	116	17 (15)	123	28 (23)
Time from seizure onset to ICU admission—median (IQR) h	109	2.5 (1.9–3.5)	116	2.7 (2.0–3.9)
First-line anti-epileptic drug—no. (%)				
Benzodiazepine^§^	117	109 (93)	124	115 (93)
Second-line anti-epileptic drugs– no. (%)	117	67 (57)	124	74 (60)
Phenobarbital	117	13 (11)	124	28 (23)
Levetiracetam	117	3 (3)	124	3 (2)
Fosphenytoin/phenytoin	117	52 (44)	124	45 (36)
Sedation—no. (%)	117	40 (34)	124	44 (35)
Midazolam	117	30 (26)	124	35 (28)
Propofol	117	4 (3)	124	3 (2)
Sodium thiopental	117	13 (11)	124	14 (11)
Interruption of seizure—no. (%)	117	102 (87)	123	107 (87)
Arousal before randomisation—no. (%)	117	10 (9)	124	13 (10)
Refractory status epilepticus—no. (%)	117	11 (9)	124	11 (9)
Mechanical ventilation—no. (%)	116	83 (72)	124	82 (66)
Glasgow coma scale—median (IQR)	112	4.0 (3.0–6.2)	117	3.0 (3.0–7.0)
SAPSII—median (IQR)	118	52 (42–61)	126	48 (38–59)
SOFA—median (IQR)	105	6.0 (5.0–8.0)	102	6.0 (4.0–7.0)
Final diagnosis—no. (%)*	118		126	
Acute symptomatic		41 (35)		46 (37)
Remote symptomatic		31 (26)		35 (28)
Progressive symptomatic		29 (25)		29 (23)
Non-epileptic spell		2 (2)		0 (0)
Other		2 (2)		2 (2)
Unknown		13 (11)		14 (11)

^§^ All the patients who did not receive a first-line ASM were sedated by either propofol or midazolam, except five who received a second-line ASM. Therefore, all the patients met the inclusion criteria

^*^ Acute symptomatic generalised convulsive status epilepticus (GCSE) occurs at the time of a systemic insult or in close temporal association with a documented brain insult; remote symptomatic GCSE occurs some years after a significant brain injury; progressive symptomatic GCSE is related to progressive neurological disorders [1].

*IQR* Interquartile range; *SAPSII* Simplified acute physiology score [36]; *SOFA* Sepsis-related Organ Failure Assessment Score [37]

### Anti-epileptic administration

**Fig. 2:**
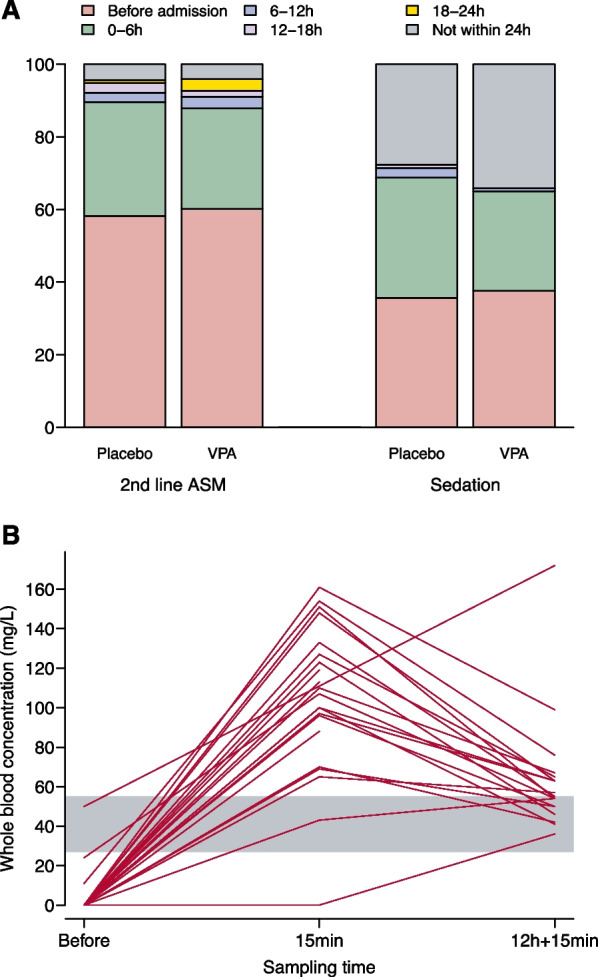
Timing of second-line anti-seizure medicine (ASM) (panel** A**) and sedation and serum concentrations of valproic acid (VPA) (panel** B**). Panel A, data were available for 241 patients (3 missing in each group) for second-line ASM (2nd-line ASM) and 229 (6 and 9 missing in the placebo and VPA groups) for sedation, by propofol, midazolam, or pentothal for at least 6 h. Even if mainly used for synchronisation with the ventilator, sedative drugs were taken into account because of their anti-epileptic properties. Panel B, whole blood concentrations are presented for the 22 VPA group participants with samples, before VPA administration (“Before”), 15 min after loading dose administration (“15 min”) and 15 min after the end of the 12-h continuous intravenous infusion (“12 h + 15 min”). The grey shaded region represents the therapeutic interval, expressed as mg/L of whole blood. One patient received VPA before hospital admission. Baseline sampling was performed after VPA administration in two patients. For note, the plasma/whole blood ratio of valproic acid is about 1.8 [40]

Eighty-three of the 103 (42%) patients who had not received a second-line ASM before being admitted to the ICU received it within the first 24 h after admission, mainly within the first 6 h (Fig. 2). From the seizure onset to the sixth hour after ICU admission, 211 (86%) received first -and second-line ASMs, including 103 (86%) and 108 (87%) patients in the placebo and VPA groups, respectively (Fig. 2). The VPA or placebo administration was started after a median duration of 35 (IQR 16 to 61) minutes after randomisation, and a median 5 (IQR 4.4 to 6.8) hours after seizure onset. In 227 (93%) patients, the second-line ASM was given before VPA or placebo infusion. Plasma VPA level reached the therapeutic range in all 22 tested patients from the VPA group (Fig. 2).

### Primary outcome

In the ITT analysis, the proportion of patients who were hospital-discharged alive at day 15 was 77/126 (61%) in the VPA group and 72/118 (61%) in the placebo group (RR: 1.04 (0.89 to 1.19),* p* = 0.58; Table 2 and Additional file 1: Fig. S1). Worst-case scenario analyses and analyses based on available data only showed similar results (Table 2). In-hospital mortality rate was 6% and 2% in the VPA and placebo groups, respectively (*p* = 0.18). Brain tumour was the main cause of death and was more frequent in the VPA group (Table 3).

**Table 2:** Primary and secondary outcomes

	Placebo (*n* = 118)	Valproic acid (*n* = 126)	Risk difference^*^ (95% CI)	Relative risk^*^ (95% CI)	P
*Primary outcome†*					
Intention to treat	72/118 (61%)	77/126 (61%)	+ 2.5% (− 6.3 to 11.2)	1.04 (0.90 to 1.20)	0.58
Worst-case scenario	73/118 (62%)	77/126 (61%)	+ 1.8% (− 7.5 to 11.1)	1.03 (0.88 to 1.20)	0.70
Available data	72/117 (62%)	77/125 (62%)	+ 2.6% (− 7.3 to 12.5)	1.04 (0.89 to 1.23)	0.60
*Secondary outcomes*					
Refractory status epilepticus	6/118 (5%)	5/126 (4%)	− 0.9% (− 5.6 to 3.8)	0.82 (0.32 to 2.08)	0.68
Super-refractory status epilepticus	4/118 (3%)	3/126 (2%)	− 1.0% (− 5.7 to 3.7)	0.71 (0.17 to 2.96)	0.64
Recurrence of seizure during ICU stay‡	14/118 (12%)	7/125 (6%)	− 6.1% (− 15.2 to 3.0)	0.48 (0.23 to 1.00)	0.049
Time to awakening (hours)	18 (9–72) [*n* = 117]	24 (12–72)			
[*n* = 121]	–	0.84 (0.64 to 1.10)^§^	0.21		
Length of ICU stay (days)	4 (3–7)	5 (3–8)	–	0.91 (0.70 to 1.18)^§^	0.46
Length of hospital stay (days)	11 (7–23)	12 (7–24)	–	0.97 (0.75 to 1.27)^§^	0.84
Highest SOFA score¶	4 (2–7) [*n* = 76]	4 (2–6) [*n* = 74]	+ 0.2 (− 0.9 to 1.4) ‖	–	0.68
Death in ICU	2/118 (2%)	4/126 (3%)	+ 1.6% (− 2.5 to 5.8)	1.99 (0.48 to 8.27)	0.35
Death in hospital	2/118 (2%)	7/126 (6%)	+ 4.0% (− 0.7 to 8.7)	2.94 (0.61 to 14.3)^§^	0.18
Death from randomisation to day 90	3/118 (3%)	8/126 (6%)	+ 4.7% (− 1.9 to 11.3)	2.32 (0.61 to 8.84)^**^	0.22
MMSE score at day 90	26 (21–29) [*n* = 21]	26 (23–28) [*n* = 18]	− 0.2 (− 4.7 to 4.2) ‖	–	0.92
FAB score at day 90	14 (12–17) [*n* = 26]	15 (12–18) [*n* = 23]	+ 0.2 (− 2.1 to 2.5) ‖	–	0.16
Glasgow Outcome Score at day 90	5 (4–5) [*n* = 44]	4 (3–5) [*n* = 55]	–	0.71 (0.33 to 1.58)^††^	–

Data are* n*/*N* (%) or median (first–third quartile) unless otherwise stated

* Adjusted for randomisation strata (age ≤ 65 or > 65 years, and presence or absence of acute brain injury), and centre

^†^ Defined by the proportion of patients discharged alive from hospital to their home or to a long-term care facility on day 15

^‡^ Defined by the recurrence of seizure or status epilepticus in patients for whom GCSE has been controlled

^§^ Adjusted sub-distribution hazard ratio

^¶^ Worst score day 2 to day 15

‖ Adjusted mean difference

^**^ Adjusted hazard ratio

^††^ Adjusted odds ratio estimated in a Bayesian proportional odds model; no P-value is computed

*CI* Confidence interval; *FAB* Frontal Assessment Battery [38]; *ICU* Intensive care unit; *MMSE* Mini-Mental State Examination [39]; *SOFA* Sepsis-related Organ Failure Assessment

**Table 3:** Safety analyses

	Placebo (*n* = 118)	Valproic acid (*n* = 126)	*P*
*Adverse events*			
Patients with at least one AE	52 (44%)	45 (36%)	0.19^*^
Patients with multiple AEs	27 (23%)	19 (15%)	
Number of AEs	102	71	0.088^†^
*Serious adverse events*			
Patients with at least one SAE	27 (23%)	33 (26%)	0.56^*^
Patients with multiple SAEs	5 (4%)	8 (6%)	
Number of SAEs	34	48	0.27^†^
Severity criteria			
Death	3	8	
Life-threatening event	6	12	
Hospitalisation	22	23	
Disability or incapacity	0	1	
Other significant event	3	4	
Related to the investigational drugs^‡^	3	0	
Serious adverse events			
Recurrence of seizure	6	8	
Recurrence of status epilepticus	4	6	
Death	2	7	
Hepatic cytolysis	3	4	
Myocardial ischaemia	1	3	
Psychogenic non-epileptic seizure	0	3	
Septicaemia	0	3	
Stroke	1	2	
Refractory status epilepticus	1	2	
Pulmonary embolism	1	1	
Shock	1	1	
Fracture	2	0	
Pneumonia	2	0	
Cardiac arrest	0	1	
Confusion	0	1	
Hemiplegia	0	1	
Hypoglycaemia	0	1	
Hyponatremia	0	1	
Acute urinary retention	0	1	
Occlusive syndrome	0	1	
Dysrhythmia	0	1	
Diarrhoea	1	0	
Encephalopathy	1	0	
Pressure ulcer	1	0	
Hyperglycaemia	1	0	
Acute renal failure	1	0	
Acute pulmonary oedema	1	0	
Valproate overdosing	1	0	
Thrombosis	1	0	
Toxidermia	1	0	
Causes of death	*n* = 3	*n* = 8	
Withdrawal of care due to severity of brain insult	2	5	
Brain tumour	0	3	
Brain metastases	1	0	
Brain tumour and stroke	0	1	
Stroke	0	1	
Dementia	1	0	
Withdrawal of care due to poor general status	0	1	
Multiple organ failure	0	1	
Withdrawal of care, reason unspecified	1	1	

Type and rates of declared adverse events occurring during hospital stay

* Fisher’s exact test

^†^ Robust Poisson model

^‡^ Assessed by the investigator

In one patient, five recurrences of seizures were each declared an adverse event (AE)

*SAE* Serious adverse event

### Secondary outcomes

The ICU, hospital, and day 90 mortality rates were similar between the two groups as well as the proportion of patients developing refractory and super-refractory status epilepticus (Table 2). At day 90, MMSE, FAB, and GOSE were assessed in 39 (16%), 49 (20%), and 99 (40%) patients and were similar between the two groups.

### Adverse events

One or more adverse events of any grade of severity were declared by the investigators during hospital stay in 52 (44%) and 45 (36%) patients from the VPA and placebo groups, respectively (*p* = 0.19, Table 3). Serious adverse events occurred in 33 (26%) patients in the VPA group and 27 (23%) patients in the placebo group (*p* = 0.56).

## Discussion

In this multicentre, double-blind, randomised, controlled, and pragmatic trial, we found that the intravenous administration of VPA, in addition to first- and second-line ASMs, did not increase the proportion of patients discharged alive from hospital within the first 15 days. We also found that VPA did not increase the occurrence of adverse events and did not decrease incidence of refractory and super-refractory status epilepticus or 90-day mortality.

The absence of impact of VPA on the patient status at day 15 could be a result of various factors. First, the observed median length of hospital stay (12 days) was close to what we had anticipated (15 days), whereas it was 21 days in the ICU-admitted GCSE population of the HYBERNATUS trial and 10 days in the pre-hospital GCSE population of the SAMUKeppra trial [9, 28]. Half of the patients in the Treiman study were discharged alive from hospital at day 30, whereas this was 61% at day 12 in our trial [29]. In comparison with the HYBERNATUS trial, the ICU length of stay was decreased by half [28]. This suggests that overall hospital care of our patients was satisfactory, resulting in a substantial reduction of ICU and hospital length of stay.

Second, the low rate of mortality and refractoriness might suggest that our cohort is not representative of ICU-admitted GCSE patients. This is unlikely given that 16 general ICUs participated in our trial. Moreover, patients from this trial were appropriately referred to the ICU, as indicated not least by the fact that there was an 85% rate of invasive mechanical ventilation within the first 24 h. It is noteworthy that only a third and one half, respectively, of GCSE patients admitted to the ICU were intubated in the ESETT and RAMPART trials [8, 30], suggesting that intubation is not the only criterion for admission to the ICU. Finally, the 5% in-hospital mortality rate is close to that reported in another French cohort of GCSE patients [9]. Our trial design allowed us to recruit de novo established GCSE patients who required admittance to a general ICU.

The main explanation for the low rate of refractory and super-refractory GCSE is that the step-by-step anti-epileptic treatment had been closely followed and was effective. Of note is the rate of refractory GCSE at ICU admission, which was 9% in our trial but 25% in the HYBERNATUS study [28]. Moreover, the first-line ASM before admission to the ICU was administered in 93% of our patients, whereas the SENSE European registry [10] states that it is commonly given in only 74% of GCSE cases. This indicates that pre-hospital care was satisfactory. Moreover, a second-line ASM was administered in more than 90% of our patients before VPA administration. These findings clearly indicate that our objective of using VPA as an adjunctive treatment to the recommended stepwise anti-epileptic strategy was attained. One could argue that the anti-epileptic treatment was excessive. However, the relatively good outcome of our patients suggests rather that anti-epileptic treatment was appropriate. Even if sedation was administered in up to 70% of our patients within the first 24 h from admission, the median times of 24 h to awakening and 3 days to extubation demonstrate that our patients were not receiving unnecessary sedative drugs. We acknowledge that continuous EEG might have been used for a more accurate diagnosis of refractoriness and guidance of the anti-epileptic treatment. However, continuous EEG was not available in most French general ICUs at the time of our study design. It is of interest to note that a standard EEG was performed in 71% of our patients within the first 24 h after their ICU admission, whilst it was achieved in only 60% of patients included in the ESETT trial [30]. EEG showed persisting epileptic activity in only 10% of cases, suggesting an effective control of GCSE in most patients. We cannot rule out that rate of refractoriness has been underestimated in our trial. On the other hand, it has been recently shown that diagnosis of refractory status epilepticus can be overestimated in patients intubated and sedated for a GCSE [31]. The total incidence of refractory GCSE (i.e. 14%) in our trial was close to the 16% reported in a retrospective cohort study of sedated and intubated GCSE patients [31]. The decision to intubate or not depends on many factors, including GCSE control, tolerability of anti-epileptic treatment, evolution of the underlying cause, and the usual practice of the physician. Therefore, intubation does not mean univocally that GCSE is not controlled, or that non-intubation unequivocally signifies regulation of GCSE.

We are not able to rule out that greater circulating levels of VPA would have allowed a better epileptic control and a greater neuroprotective effect, as evidenced in experiment models [32, 33]. It might also induce more frequent or more severe side-effects. The delay of six hours between seizure onset and VPA infusion can be considered too long and that an earlier (notably during pre-hospital care) administration VPA might have been efficient on the primary outcome. We also acknowledge that non-clinical seizures were likely not detected by a single standard EEG. In addition, the fact that the cognitive status has been assessed in only a third of our cohort hampers to draw any conclusion on VPA neuroprotective effect. Heterogeneity of our population might be considered a limitation to the results of this trial. As with a previous cohort of ICU-admitted GCSE patients [28], our population is heterogeneous in terms of aetiology, GCSE severity, time course, and prognosis factors and pre-ICU management. A homogeneous population incorporating all these criteria is unattainable. Moreover, we rather think that heterogeneity is an advantage for the generalisability of our results. First, our pragmatic trial is representative of GCSE patients and their treatment in general ICUs. We have attempted to reduce the heterogeneity by stratifying randomisation on major predictors, such as age and presence of acute brain injury, and by standardising the first-line and second-line anti-epileptic treatments. GCSE is a dynamic process and patients are not all admitted to the ICU at the same time point in the course of GCSE. As proposed in previous trials on ICU-admitted GCSE patients [28, 34], restricting the inclusion to only GCSE patients who were intubated or were still seizing at time of their admission to the ICU is questionable. As stated previously, a decision to intubate is dependent on various factors and often made before admission to the ICU [31]. The absence of clinical seizure in comatose patients referred to an ICU for GCSE—which was the case in most of those in our study—does not imply that the epileptic process is controlled. Basing inclusion on EEG features would not have been feasible and generalisable as EEG cannot be rapidly performed in most general ICUs, at least in France, and also because the complexity and delay of EEG interpretation is a consideration [1]. For this reason, it is noteworthy that the EEG-guided Treatment of Refractory Status Epilepticus trial on the use of propofol versus barbiturates was prematurely terminated because of insufficient recruitment [34]. Moreover, randomisation has enabled us to satisfactorily balance factors of heterogeneity between our two therapeutic groups, making the results interpretable. Finally, our results concern only patients who had no contraindication to VPA, and therefore exclude women of childbearing age. VALSE would warrant a further trial testing a different adjuvant ASM in these patients.

Another limitation is related to the early termination of the trial. Although 99% (245/248) of the calculated sample size had been enrolled, which would have led to negligible loss of power, some participants did not receive the allocated treatment and a few outcomes were missing, so that the power of the trial may be slightly decreased. However, the confidence interval of the treatment difference on the primary outcome clearly ruled out the anticipated 20% risk reduction.

In conclusion, administration of VPA, when added to the recommended stepwise anti-epileptic regimen, is well tolerated but not associated with a significant impact on hospital discharge or evolution towards refractory or super-refractory status epilepticus in patients admitted to an ICU for GCSE. Altogether, our results indicate that most ICU-admitted GCSE patients were treated according to available guidelines and had good short-term outcomes.

## Supplementary Information

**Additional file 1.** Supplementary appendix.

## Data Availability

Data available: Yes. Data types: De-identified participant data. How to access data: De-identified patient data to reproduce results presented in the article when available: With publication Supporting Documents Document types: None. Who can access the data: Researchers whose proposed use of the data has been approved. Types of analyses: Research projects with the same scientific purpose as the original study (treatment of myasthenia gravis), such as meta-analysis, for instance. Mechanisms of data availability: Data will be made available upon approval of a proposal, Data will be made available upon approval of a proposal, authorization from the French Comité de Protection des Personnes (IRB) who authorized the study, and after a signed data access agreement with the trial sponsor. Any additional restrictions: none.

## Abbreviations

ASM: Anti-seizure medicine
FAB: Frontal assessment battery
GCSE: Generalised convulsive status epilepticus
GOSE: Glasgow outcome scale-extended
ICU: Intensive care unit
IQR: Interquartile range
ITT: Intention to treat
MMSE: Mini-mental state examination
RR: Relative risk
SAPSII: Simplified acute physiology score simplified acute physiology score
SOFA: Sepsis-related organ failure assessment
VPA: Valproic acid
