# Supplementary material for: Valproic acid as adjuvant treatment for convulsive status epilepticus: a randomised clinical trial
Source: Crit Care. 2023 Jan 9;27:8. doi: 10.1186/s13054-022-04292-7 (PMC9830759; doi:10.1186/s13054-022-04292-7)
Supplement: Supplementary file 1 — Additional file 1. Supplementary appendix. [file 13054_2022_4292_MOESM1_ESM.docx]

**SUPPLEMENTARY APPENDIX**

**Methods**

**Study design:** A steering committee provided guidance throughout the trial. An independent data and safety monitoring board (DSMB) monitored the safety data. The trial was investigator-initiated, and there was no industry support or involvement. The investigators were responsible for the trial design, data collection, and data analysis.

**Informed consent:** If the patient presented impaired consciousness, the investigator sought written consent from the next of kin. If the latter was not present, the patient could still be included as deferred consent has been approved by the ethics committee, according to French law (Art L1122-1-2 du Code de la Santé Publique). As soon as the patient’s status allowed, written informed consent was obtained for the continuation of the research and data analyses.

**Inclusion criteriae**: It was originally permissible to include patients with recurring or persisting GCSE despite anti-epileptic treatment within the last 24 hours*.* In fact, only twelve patients fell into this category, indicating that we have included patients admitted to the ICU for *de novo* GCSE of early evolution and for which anti-epileptic treatment had been rapidly initiated.

**Randomisation and interventions**

**Administration of VPA:** VPA treatment consisted of intravenous administration of a loading dose of 30 mg/kg over 15 minutes followed by a continuous intravenous dose of 1 mg/kg/h over the next 12 hours, in accordance with the practice and recommendations later proposed by Trinka and colleagues.(1)

**Non convulsive status epilepticus**: We anticipated that a generalised convulsive status epilepticus (GCSE) can progressed to non-convulsive SE with persistent altered mental status (also called subtle status epilepticus). We included only GCSE, of which onset was seizure.

We acknowledged that persistent altered mental status can be related to other causes than persistent epileptic process, such as: 1) an effect of the second-line anti-epileptic drug, which has been shown to shorten the delay for return of consciousness(2); effect of the underlying brain lesion. Only an EEG could determine whether the epileptic process is persisting or not. Unfortunately, EEG cannot be rapidly performed as part of the routine in most of French ICUs. We then reasoned that in case of persisting altered mental status the possibility of an uncontrolled status epilepticus should prevail, if an EEG cannot be very rapidly performed for ruling it out. Our trial favoured a pragmatic management.

**Outcomes and assessment**

**Primary endpoint:** Hospitalisation lasting more than 15 days was not considered a failure if the patient was declared fit for discharge from hospital but remained hospitalised because of social issues or a lack of bed availability in recovery facilities.

It must be emphasised that the primary endpoint varies across clinical trials. The primary endpoint of multicentre randomised clinical trials on pre-hospital treatment has mostly been the control of clinical seizure. (3–5) We considered that progress to refractory or super-refractory GCSE would not be an appropriate primary endpoint because it would have required use of continuous electroencephalogram (EEG), which was not available in most general ICUs at the time of our study design. As a matter of fact, an EEG-based clinical trial on refractory GCSE was prematurely interrupted because of insufficient recruitment. (6) We acknowledge that long-term neurological status could have been an option. For instance, the HYBERNATUS trial has assessed the Glasgow Outcome Scale (GOS) at three months(7). However, long-term neurological status was shown to depend on age and aetiology of GCSE rather than on GCSE alone. (8) Moreover, the neurological assessment should not rely only on the GOS but should also include a face-to-face interview with patients. However, we anticipated that such a follow-up would be associated with a high risk of loss of follow-up, which was later confirmed by the HYBERNATUS trial, which reported that only 22% of the GCSE patients underwent a face-to-face evaluation. (7) For all these reasons, we considered that the primary endpoint should be for a shorter outcome, determined to some extent by control of the epileptic process. The clinical status at day 15 is an objective and clinically relevant primary endpoint that reflects the efficacy of overall care of ICU-admitted GCSE patients, including the control of seizure. In 1998, Treiman and colleagues reported that 50% of patients with overt GCSE would be discharged at day 30. (4) We anticipated that in 15 years this proportion of patients discharged alive would be achieved earlier, at day 15. The length of hospital stay was, respectively 11 and 20 days for non-refractory and refractory GCSE, i.e., 15 days on average. (9)

The maintenance ASMs were recorded. Standard biological tests were performed upon prescription by the ICU physician in charge of the patients.

**Results**

**Characteristics of the patients**: The anti-epileptic treatment was initiated less than six hours before inclusion in all but twelve patients.

117 (49%) patients had a history of epilepsy, and 109 (45%) had received a pre-existing anti-epileptic treatment. Sixty percent of our patients were referred by MICUs, 32% from emergency departments and 8% from other hospital wards.

Eighty-four patients (34%) were sedated at time of ICU admission.

Two patients (0.8%) were secondarily diagnosed as psychogenic non-epileptic seizure.

There were two main reasons of the absence of EEG in 29% of the patients, withing the 24 hours from their admission in ICU. First, EEG was thought not useful in patients who had clinically recovered from their GCSE. The proportion of patients who had awaken within the first 24 hours was lower, although not statistically significant, among patients who underwent EEG than those who did not (51% versus 61%, p=0.36). Second, EEG was not available. In addition, EEG is often not available after 6 PM and during the weekend in many centres. The highly specialized neuro-ICU are rather solicited for managing super-refractory GCSE.

It is interesting to note that less patients were discharged alive at day 15 (i.e., primary endpoint) among patients with EEG than those without EEG within the first 24 hours (58% versus 72%).

The proportions of patients who underwent brain imaging and lumbar puncture were comparable between the two groups (i.e., 81% versus 75%; 14% versus 18%, respectively, Table S2 of supplementary appendix).

For six patients, the assigned treatment (i.e., VPA) was not available in the pharmacy. In four patients, the physician deemed that the patients have been mistakenly included because they were all found after inclusion to be under guardianship.

**Primary outcome**: No patient had hospital discharge deferred beyond day 15 for non-medical reasons.

**Management of persisting EEG seizure within the first 24 hours:** The management of the 17 patients with persisting seizure on EEG prompted to start or increase midazolam or propofol in all the patients in association with a new ASM in 10 patients and thiopental in two patients.

**Maintenance ASM**: The proportion of patients who received a maintenance ASM within the first 48 hours was similar between the two groups (75 [75%] in the placebo group versus 78 [79%] in the VPA group; Table S3 supplementary appendix).

**Secondary outcomes**: The recurrence of seizure (i.e., defined by the recurrence of seizure or status epilepticus in patients for whom GCSE had been controlled) was significantly less frequent in the VPA cohort than in the placebo group (6% versus 12%, p=0·049, RR: 0·48 [0·13 to 0·83]).

**Adverse events**: Twenty-four recurrences of seizure were declared as adverse events, 21 (88%) having occurred after ICU discharge. In one patient, five recurrences of seizures were each declared as an adverse event. Hepatic cytolysis was reported in 4 and 3 patients of the VPA and placebo groups, respectively.

**Discussion**

The reduction in the recurrence of seizure during ICU stay must be viewed with caution. If the rate of the recurrence of seizure was similar to that reported in previous GCSE trials(3, 5, 7), it is relatively low, and its reduction appears to have no global impact on the trajectory of GCSE subjects whilst risking exposing patients to additional ASM with little benefit. Moreover, the increase in mortality, even if not significant, must make us wary about the use of VPA.

Therefore, the absence of impact on the patient status at day 15 suggests that the latter does not depend on the control of GCSE but on other factors such as aetiological investigation, optimisation of the maintenance ASMs, and organisation of hospital discharge. These factors were not within the scope of our trial.

One argument for using VPA was its potential neuroprotective effect, but the absence of effect on the long-term cognitive and functional status does not support this hypothesis. However, the large number of patients lost to follow-up precludes any definitive conclusion. Our study highlights the difficulty of seeing these patients at an outpatient clinic for cognitive assessment after ICU discharge, as has been reported in previous studies, and it was one of the reasons we did not use long-term outcomes as a primary endpoint.(7)

EEG was not performed in third of our patients, within the first 24 hours from their admission. Even if less patients were discharged alive at day 15 (i.e., primary endpoint) among patients with EEG than those without EEG within the first 24 hours, this should not deter the ICU-physician to request an EEG. we are convinced that it would have been better that EEG was performed. It would have helped to better characterize the course of the GCSE.

We would like to remind that a standard EEG was performed within the 24 hours from their admission in only 60% in the ESETT trial(10). In addition, EEG interpretation can be also complex and time-demanding (11). For instance, the mean delay for interpretation of continuous EEG was 6.6 hours in the HYBERNATUS trial, which is quite delayed given that the patients could be included up to 8 hours after the onset of seizures(7).

One may argue that we should have only included only patients with at least refractory status epilepticus Limiting inclusion to refractory GCSE is also problematic. The propofol versus barbiturates trial was interrupted because of recruitment failure(6). The proportion of patients with refractory GCSE was 25% in the HYBERNATUS trial but was only 9% in our trial. As mentioned previously, an accurate assessment of refractoriness required an EEG, which is not easily available in most general ICUs. Moreover, EEG interpretation can itself be problematic and varies among centres. As noted by Trinka and colleagues(11), there are “no-evidence-based EEG criteria for status epilepticus”. The mean delay for interpretation of continuous EEG was 6.6 hours in the HYBERNATUS trial, which is quite delayed given that the patients could be included up to 8 hours after the onset of seizures(7).

In addition to the difficulty of assessing refractoriness, we thought that excluding patients with non-refractory GCSE wrongly presumed that these patients could not subsequently deteriorate. VPA was chosen as an adjuvant therapy for potentializing the control of GCSE and for its potential neuroprotective properties.

We acknowledge that having included non-seizing and conscious GCSE patients might seem disproportionate. We originally thought VPA could improve their outcome because of its anti-epileptic and neuroprotective properties, especially as it is also well-tolerated. These patients account for less than 10% (n=23) of our cohort. Out of those 23 patients, it must be noted that 8 were intubated within 24 hours of ICU admission (30%).

**References**

1. Trinka E, Höfler J, Zerbs A, Brigo F. Efficacy and Safety of Intravenous Valproate for Status Epilepticus: A Systematic Review. *CNS Drugs* 2014;28:623–639.

2. Kämppi L, Ritvanen J, Mustonen H, Soinila S. Delays and Factors Related to Cessation of Generalized Convulsive Status Epilepticus. *Epilepsy Res Treat* 2015;2015:591279.

3. Navarro V, Dagron C, Elie C, Lamhaut L, Demeret S, Urien S, An K, Bolgert F, Tréluyer J-M, Baulac M, Carli P. Prehospital treatment with levetiracetam plus clonazepam or placebo plus clonazepam in status epilepticus (SAMUKeppra): a randomised, double-blind, phase 3 trial. *The Lancet Neurology* 2016;15:47–55.

4. Treiman DM, Meyers PD, Walton NY, Collins JF, Colling C, Rowan AJ, Handforth A, Faught E, Calabrese VP, Uthman BM, Ramsay RE, Mamdani MB. A comparison of four treatments for generalized convulsive status epilepticus. Veterans Affairs Status Epilepticus Cooperative Study Group. *N Engl J Med* 1998;339:792–798.

5. Kapur J, Elm J, Chamberlain JM, Barsan W, Cloyd J, Lowenstein D, Shinnar S, Conwit R, Meinzer C, Cock H, Fountain N, Connor JT, Silbergleit R. Randomized Trial of Three Anticonvulsant Medications for Status Epilepticus. *New England Journal of Medicine* 2019;381:2103–2113.

6. Rossetti AO, Milligan TA, Vulliémoz S, Michaelides C, Bertschi M, Lee JW. A randomized trial for the treatment of refractory status epilepticus. *Neurocrit Care* 2011;14:4–10.

7. Legriel S, Lemiale V, Schenck M, Chelly J, Laurent V, Daviaud F, Srairi M, Hamdi A, Geri G, Rossignol T, Hilly-Ginoux J, Boisramé-Helms J, Louart B, Malissin I, Mongardon N, Planquette B, Thirion M, Merceron S, Canet E, Pico F, Tran-Dinh Y-R, Bedos J-P, Azoulay E, Resche-Rigon M, Cariou A. Hypothermia for Neuroprotection in Convulsive Status Epilepticus. *New England Journal of Medicine* 2016;375:2457–2467.

8. Legriel S, Azoulay E, Resche-Rigon M, Lemiale V, Mourvillier B, Kouatchet A, Troché G, Wolf M, Galliot R, Dessertaine G, Combaux D, Jacobs F, Beuret P, Megarbane B, Carli P, Lambert Y, Bruneel F, Bedos J-P. Functional outcome after convulsive status epilepticus. *Crit Care Med* 2010;38:2295–2303.

9. Novy J, Logroscino G, Rossetti AO. Refractory status epilepticus: a prospective observational study. *Epilepsia* 2010;51:251–256.

10. Kapur J, Elm J, Chamberlain JM, Barsan W, Cloyd J, Lowenstein D, Shinnar S, Conwit R, Meinzer C, Cock H, Fountain N, Connor JT, Silbergleit R, NETT and PECARN Investigators. Randomized Trial of Three Anticonvulsant Medications for Status Epilepticus. *N Engl J Med* 2019;381:2103–2113.

11. Trinka E, Cock H, Hesdorffer D, Rossetti AO, Scheffer IE, Shinnar S, Shorvon S, Lowenstein DH. A definition and classification of status epilepticus – Report of the ILAE Task Force on Classification of Status Epilepticus. *Epilepsia* 2015;56:1515–1523.

**Table S1. Detailed aetiology of generalised convulsive status epilepticus (GCSE)**. Data were available for 236 (97%) participants.

| **Causes of status epilepticus^*^** | **Placebo (n=115)** | **Valproic acid (n=121)** |
| --- | --- | --- |
| Acute cerebral lesion | 21 (18) | 24 (20) |
| *Ischaemic stroke* | *4 (4)* | *8 (7)* |
| *Haemorrhagic stroke* | *2 (2)* | *1 (1)* |
| *Cerebral venous thrombosis* | *1 (1)* | *2 (2)* |
| *Traumatic brain injury* | *3 (3)* | *0 (0)* |
| *Central nervous system infection* | *4 (4)* | *2 (2)* |
| *Brain tumour* | *6 (5)* | *10 (8)* |
| *Other* | *1 (1)* | *3 (3)* |
| Progressive cerebral lesion | 6 (5) | 5 (4) |
| *Related to a genetic disorder* | *0 (1)* | *1 (1)* |
| *Related to a degenerative disease* | *2 (2)* | *2 (2)* |
| *Other* | *4 (4)* | *2 (2)* |
| Sequelae of a remote cerebral lesion | 26 (23) | 29 (24) |
| *Related to stroke* | *14 (12)* | *14 (12)* |
| *Related to traumatic brain injury* | *6 (5)* | *7 (6)* |
| *Related to neurosurgery* | *5 (4)* | *3 (3)* |
| *Related to central nervous system infection* | *2 (2)* | *1 (1)* |
| *Other* | *2 (2)* | *6 (5)* |
| Systemic disorder | 4 (4) | 7 (6) |
| *Metabolic disorder: hyponatraemia* | *2 (2)* | *1 (1)* |
| *Systemic infection* | *1 (1)* | *0 (0)* |
| *Other systemic disorder* | *2 (2)* | *6 (5)* |
| Drug-related or poisoning | 18 (16) | 18 (15) |
| *Underdosing of anti-epileptic drug* | *7 (6)* | *8 (7)* |
| *Adverse drug rection* | *3 (3)* | *2 (2)* |
| *Medication withdrawal* | *4 (4)* | *2 (2)* |
| *Alcohol abuse* | *3 (3)* | *3 (3)* |
| *Alcohol withdrawal* | *5 (4)* | *3 (3)* |
| *Recreative drug use* | *1 (1)* | *4 (3)* |
| Neurologic spell | 2 (2) | 0 (0) |

* One patient may have more than one reported aetiology

Data are numbers (percent).

**Table S2. Aetiological investigations at ICU admission.** Data were available for 237 (97%) participants.

| **Investigation** | **Placebo (n=115)** | **Valproic acid (n=122)** |
| --- | --- | --- |
| Computed tomography | 93 (81) | 92 (75) |
| Magnetic resonance imaging | 6 (5) | 9 (7) |
| Unspecified imaging | 1 (1) | 0 (0) |
| Cerebrospinal fluid testing | 40 (35) | 43 (35) |
| Anti-epileptic drugs dosage | 416/113(14) | 22 (18) |

Data are numbers (percent).

**Table S3. Maintenance anti-epileptic drug within the first 48 hours.** Data were available for 199 (82%) participants.

| **Treatment** | **Placebo (n=100)** | **Valproic acid (n=99)** |
| --- | --- | --- |
| Any maintenance drug | 75 (75) | 78 (79) |
| Valproic acid | 6 (6) | 6 (6) |
| Phenobarbital | 6 (6) | 14 (14) |
| Clobazam | 34 (34) | 40 (40) |
| Levetiracetam | 29 (29) | 20 (20) |
| Lamotrigine | 7 (7) | 18 (18) |
| Phenytoin | 4 (4) | 9 (9) |
| Lacosamide | 8 (8) | 5 (5) |
| Diazepam | 2 (2) | 5 (5) |
| Carbamazepine | 5 (5) | 2 (2) |
| Gabapentin | 3 (3) | 0 (0) |
| Oxcarbazepine | 1 (1) | 2 (2) |
| Clonazepam | 0 (0) | 1 (1) |
| Other | 2 (2) | 1 (1) |

Data are numbers (percent).

**Figure S1. Distribution of time to discharge and in-hospital death over follow-up.** The figure displays the cumulative incidence of both events.
